# An engagement framework for the authentic co-design of a consent and healthy relationships intervention with upper-secondary students

**DOI:** 10.3389/frph.2024.1420895

**Published:** 2024-09-13

**Authors:** Ashleigh M. Pantaleo, Peta L. Dzidic, Elizabeth Newnham, HuiJun Chih, Robert Wells, Brad Olson, Sarah Langley, Adrian Schonfeld, Jacqueline Hendriks

**Affiliations:** ^1^ Curtin School of Population Health, Curtin University, Bentley, WA, Australia; ^2^ Curtin Gender Research Network, Curtin University, Bentley, WA, Australia; ^3^ Curtin EnAble Institute, Curtin University, Bentley, WA, Australia; ^4^ FXB Center for Health and Human Rights, Harvard University, Boston, MA, United States; ^5^ School of Allied Health, Curtin University, Bentley, WA, Australia; ^6^ College of Psychology and Behavioral Sciences, National Louis University, Chicago, IL, United States; ^7^ Faculty of Religion, Philosophy and Ethics, Guildford Grammar School, Guildford, WA, Australia; ^8^ Hale School, Student Wellbeing Team, Wembley Downs, WA, Australia

**Keywords:** co-design, participatory action research, consent, healthy relationships, secondary education

## Abstract

**Introduction:**

The objective of this demand driven research is to co-design an intervention for upper-secondary students that addresses issues of consent and healthy relationships. In this paper, we (university researchers, student co-researchers, school staff), present the engagement framework that has been critical to the project's development and planned implementation.

**Methods:**

An iterative co-design approach grounded in a participatory research approach is currently being adopted. Student co-researchers from three independent secondary schools on Whadjuk Nyungar Country in Boorloo/Perth, Western Australia, have been engaged as co-researchers in the design of the intervention. Supplementary quantitative and qualitative data from students enrolled at each school site is also being collated to further inform the intervention design. Student co-researchers will provide insights on the overarching design of the intervention including: the scope of key concepts they want to learn, interpretation of supplementary data, and the development of contextually relevant educative content.

**Results:**

Retrospective and prospective components of the engagement framework are described and supported with applied examples where applicable. Preliminary results demonstrate the imperative of adopting iterative co-design, and the efficacy of our authentic engagement framework. A draft intervention has been formalized and will soon undergo piloting. The co-design process has already resulted in an intervention that differs from the initial program first conceptualized by university researchers.

**Conclusion:**

Imperative to our engagement framework is centering students as experts of their lived experience. It is anticipated that this engagement framework will provide insights around the feasibility, value, and necessity for authentic engagement of upper-secondary school students in the design of their consent and healthy relationship education.

## Introduction

There is growing acceptance and acknowledgement that adolescents and young people have the ability to shape policy, programs and systems that impact their development and health (1, 2), and that different degrees of participatory engagement may be possible (3). Furthermore, the United Nations states that a child can freely give their views on issues that directly affect them (4). An integrative review of international programs that consulted with youth demonstrated positive outcomes for participants, social service organizations and communities (5). Similarly, a systematic review of youth consultation in the United States reported increased levels of agency and leadership, and improvements to various social, emotional and interpersonal outcomes when young people have been genuinely consulted (6).

In the context of sexual and reproductive health, there is acknowledgement that youth voice is not routinely considered. Villa-Torres and Svanemyr (7) have systemically critiqued that youth participation in the sexual and reproductive health sector is lacking, advocating strongly for the rights of young people to be involved in program and policy development. Furthermore, in the context of sexuality education, the views and considerations of young people are often not routinely heard, and they are afforded little to no input into the development of relevant materials (8, 9). This occurs despite international technical guidance that clearly advocates for a learner-centered approach and youth input when designing and implementing school-based sexuality education programs (10).

The need for contextually relevant comprehensive sexuality education (CSE) within the Australian school system has never been more important. Results from the *7th National Survey of Secondary Students and Sexual Health* acknowledge this necessity. Despite an expressed desire for CSE to be engaging and relevant, the 6,841students in this survey felt current school-based programs were largely irrelevant to their needs (11). Furthermore, Australian secondary school students have also called for affirming and age-appropriate CSE content (12).

Against this backdrop of student perception, primary prevention activities seeking to address sexual violence against women and children have become an area of recent focus in Australia (13). This is due to greater awareness surrounding the level of unwanted sex and sexual violence experienced by young people whilst at secondary school (11), university (14), and in the workplace (15). Similarly, the recent *Australian Child Maltreatment Study* reported high rates of harmful sexual behavior expressed by young people, including increased levels of peer-on-peer offences (16). In most instances, males are the perpetrators of this violence and females the victim/survivors, but all gender dynamics are possible. Whilst various action plans and interventions are currently underway, a national stocktake and gap analysis of respectful relationships education programs in Australia clearly identified a lack of youth voice in the development of recent programs (17).

To compound these issues, throughout most states and territories of Australia, there is presently no requirement for students to receive health education or any content related to consent, healthy relationships or sexual health during the last two years of secondary school (i.e., grades 11 and 12; student typically aged 16–18 years) (18). This results in wide variability in the way these issues may be addressed within classrooms across the country (17) at a time when many young people are expressing an interest in or engaging in various intimate and sexual encounters (11).

Whilst co-research approaches such as Participatory Action Research (19) and Youth Participatory Action Research [YPAR (20)]; may be valued by researchers, a recent scoping review identified that few Australian studies use co-research approaches in the context of adolescent sexual and reproductive health (21). As such, this paper outlines the participatory engagement framework that we (university researchers, student co-researchers, school staff), are using as part of a recently funded health promotion intervention located on Whadjuk Nyungar Country in Boorloo/Perth, Western Australia. The intention of this collaboration is to co-design an intervention for upper-secondary students that addresses issues of healthy relationships and consent in a contextually relevant manner. The planned approach is outlined, alongside recent amendments that have been incorporated due to the reflexive and iterative nature of this research method.

## Materials and methods

### Methodology

As a research team we approached this co-design initiative with a social constructivist lens, utilizing PAR. A social constructivist approach understands knowledge as socially-tethered and derived from the interactions of people, across and within groups, within a broader context (22, 23). PAR is an investigative approach which values the lived-experience of co-researchers, and which seeks to develop understanding through the integration of shared perspectives from multiple groups who are perceived to hold relevant academic, and/or lived-experience knowledge (21). While a range of philosophical theories can be applied to participatory approaches, Anyon et al. (6) indicate that it is well aligned with social constructivism in that both value the interactions between people who carry socially-derived and contextually-relevant experiences.

The key contributing factors to successful implementation of a consent and healthy relationships intervention for upper-secondary students are identified as: (a) current guidance provided by relevant local and national school curriculum and other guiding frameworks (e.g., *Western Australian Curriculum*, *Australian Student Wellbeing Framework*, *Western Australian Equal Opportunity Act 1984),* (b) national and international evidence related to best-practice approaches of relevance to the intervention, (c) participants’ and co-researchers’ perspectives of what makes such an intervention fit-for-purpose, and (d) the socially-derived needs of students who participate in the intervention. As such, each of the identified partners in this participatory process (university researchers, student co-researchers, school staff) are considered to hold knowledge and insight that is vital to the successful development and implementation of this intervention, and purposeful collaboration will be critical.

### Project inception and recruitment of school sites

The project stemmed from discussions among the research team and secondary school staff following participation in the *March4Justice* held in 2020. The March reflected a grassroots call for equality and justice, and an end to gender-based violence in Australia. The resulting grant proposal was demand driven, as a particular secondary school initially approached Curtin University staff seeking support to develop contextually relevant and appropriate sexuality education, primarily for their upper-secondary school students. Once funding was secured, two additional schools were purposively approached to participate as pilot sites. These schools were already professionally connected to some of the university researchers. This has resulted in the recruitment of three schools reflecting both single sex and mixed-sex student populations. All schools belong to the Association of Independent Schools Western Australia.

### Recruitment of school staff and student co-researchers

For each school site, a staff member was identified to act as a liaison between their respective school and Curtin University. These staff do not actively participate in data collection activities but are a valued source of information regarding the policy and procedures of their respective schools. They also provide expert knowledge of their school's students, identify potential student co-researchers, and provide ongoing support to these students throughout the research period.

Each school site under direction of the school staff member, issued a wide recruitment call to all students who would be enrolled in Year 11 (aged approximately 16–17 years) during 2023, seeking their interest to be student co-researchers. The intention was for three to five student co-researchers from each school to be identified. Qualitative research guidelines support homogenous groups of approximately 6–12 informants (24).

### Data collection

#### Initial scoping and informal meetings with student co-researchers

Students who expressed an interest to be student co-researchers were invited to individual informal meetings that included the school liaison and two of the university researchers. This informal meeting served multiple purposes. Initially, it enabled the beginnings of rapport building between parties and to try and break down any power dynamics. Secondly, it enabled the university researchers to establish why the potential student co-researchers were motivated to participate and if there were any particular outcomes that they wanted from experience.

Given a participatory approach aims to challenge traditional research power dynamics (25), this process of reflection requires acknowledgement that whilst we require something from the student co-researchers (i.e., assistance to co-design a contextually relevant intervention) we must simultaneously consider how we can give back to the co-researchers. These informal meetings enabled reciprocity through the determination of potential skills as well as areas of specific interest and experiential development. For example, there is an opportunity for co-researchers to gain insight and experience in participating in co-research, intervention development, and knowledge dissemination activities.

Finally, this informal meeting process also provides the student with an “out”. It is key that participation by co-researchers is completely voluntary (25). Research teams need to account for the possibility that students may be pressured into participating by external parties, therefore, an informal meeting enables researchers to clearly describe the scope of work and expectations, and to unpack student motivations for participation. The students interviewed across the three participating schools expressed genuine interest and drive to participate, however, if this was not the case, we were able to exclude the student(s), at their request, whilst acknowledging the student has actively inquired about participating, giving them a socially safe option to withdraw.

#### Student co-researcher consultations and intervention development

The process of engagement with the student co-researchers is iterative, and the collaborative development of the intervention involves multiple workshops. The initial workshop at each school site was to welcome and introduce all parties, and to set the scene. Effort was made to provide a casual environment with a flat organizational structure, where all participants could make an equal contribution. Workshops were conducted separately at each school to ensure an environment conducive to open communication. The process of PAR was briefly summarized, and there was acknowledgement that whilst effort would be made to genuinely and authentically engage with student co-researchers throughout all the ongoing phases of the research process, there was the potential that particular suggestions or requests could not be accommodated for various reasons. For example, the university researchers, school staff or schools may need to consider financial, time or resource constraints. Further, it was emphasized that while students’ expertise would inform the intervention, the research team were responsible for creating materials and resources, to reduce the burden on student co-researchers.

A variety of potential interventions and activities were proposed, to serve as a foundational start to the planning process and these were discussed during early workshops. Subsequent workshops involved presenting the co-researchers with draft versions of the lessons and asking for feedback regarding overall structure and specific activities within each lesson. Some feedback arose through predetermined questions that arose during the writing of the lessons. For example, what sorts of case studies or vignettes should be discussed or role-played. Other feedback was extemporaneous and came about through group discussion amongst the co-researchers about the lessons and activities proposed.

Formal notes were taken by members of the research team who were in attendance and any notations that students made were also collected with permission. Parallel to the workshops, student co-researchers reflected upon the supplementary quantitative and data that had been provided by their peers (see below) to help frame their recommendations. At each school site, up to two 60–90 min workshops were planned, dependent on the availability of the student co-researchers and school staff. Any information received in one workshop was fed forward to the next school and workshop session, so all co-researchers were aware of what other schools and co-researchers were reporting.

Prospectively, there is the intention to bring all the student co-researchers together for one final workshop that will present the intervention in its entirety prior to it being piloted by each of the schools. Due to the staggered nature of the school recruitment this event had to be offered towards the end of the planning phase. In this final workshop, the student co-researchers will be asked about their preferred definition of a “successful” outcome of the pilot program by agreeing to a list of formative pre-post mixed methods evaluation tools that address the key aspects of this “success”. They will also provide feedback on the appropriate length of the measures in addition to the phrasing of the evaluation questions.

The research team expects logistical challenges in this endeavor to bring all students together, given the constraints and independent nature of the school's structure and schedules, and the realities of working in an authentic cross-institutional participatory space. School constraints are a known challenge when conducting participatory research with adolescents [see (26)]. Whilst acknowledging this potential challenge, it is a critical component of our co-research process to reflect and integrate multiple voices across the varied independent schools’ contexts. Scheduling meetings with co-researchers in the later parts of high-school education across multiple schools is likely to be the most pressing challenge.

#### Supplementary quantitative and qualitative data from student peers

Concurrent to engaging with student co-researchers at each school site, all students in grades 10–12 (students typically aged 15–18 years) were provided with the opportunity to answer a brief online survey. This instrument was designed initially by the university researchers, with input from the student co-researchers from the first recruited school. The survey was hosted on the Qualtrics platform, and the estimated completion time was 10–15 min. In total, the survey asked 15 closed and open-ended questions that sought to explore (I) current learning experiences related to consent and healthy relationships, (II) utility of current learning, (III) preferred sources of information, and (IV) preferences for future learning (e.g., content, mode of delivery, facilitator preferences).

At the end of the survey, respondents could also elect to participate in an additional online interview with two members of the research team so they could elaborate on these same issues. These interviews were conducted online via Microsoft Teams, lasted up to 45 min, and were transcribed via the transcription platform Otter. The student co-researchers were given access to all qualitative and quantitative data so that they could integrate these findings into any recommendations that they made.

#### Evaluation of the intervention

All parties will also be involved in the development of the evaluation framework. A variety of potential items and relevant validated measures will be chosen to establish baseline levels and to measure the impact of the intervention. Process and short-term impact data will be collated. Observational notes will also be taken during the pilot phase. Student co-researchers will be consulted to determine what they consider to be a sign of intervention “success.” It is hypothesized that there will be significant changes in the outcomes prior to and after completing the workshop. Subgroup analyses may be performed for students of different gender, ethnicity, school type, should there be sufficient responses.

#### Interviews with student co-researchers

Prospectively, the final phase of data collection will be interviews with the co-researchers to explore their experiences in engaging in this co-research process. The main questions within the semi-structured interviews will focus on (I) positive aspects of the process, (II) negative aspects of the process, (III) suggestions to improve the process, and (IV) their suggestions for how to engage future student co-researchers from other secondary schools, including students who may express different cultural characteristics to themselves.

The interviews will either be conducted face to face or online via Microsoft Teams. Interviews will be transcribed via Otter at which time the transcripts will be reviewed for accuracy to ensure the full interview is captured. The transcripts will be analyzed using Reflective Thematic Analysis (22) employing both manual and NVivo inductive coding. Member checking will be adopted to develop confidence in the findings and to ensure co-researcher feedback is interpreted accurately (27).

### Ethical considerations

Ethics approval for this project was granted by the Curtin Human Research Ethics Committee (HRE2022-0382). The ethics approval is subject to ongoing amendments, as new initiatives are delivered or as the protocol is amended by the student co-researchers. For example, given the iterative nature of the research, more feedback was warranted from co-researchers, so the research team sought ethical amendments to enable additional co-researcher workshops. Furthermore, once the intervention is finalized, an amendment will be submitted to cover the piloting phase and associated evaluation activities.

At present, all prospective schools and student co-researchers have been provided with a Participant Information Sheet that clearly outlines the project, what level of involvement is required from them, what data will be collected and how this information will be stored. Written consent was obtained from schools, student co-researchers, and a parent/guardian. Any student in grades 10–12 who participated in either the online survey or interview was also provided with a Participant Information Sheet and provided written consent. Their parent/guardian was only required to provide consent for students participating in an interview.

### Current status

At the time of publication, 13 co-researchers (16–18 years, 5 female, 8 male) have been recruited, across the three participating schools. Furthermore, lesson plans have been drafted and all co-researchers have provided extensive feedback regarding various proposed activities. We have also ascertained the range of skills, opportunities and activities that co-researchers would like to achieve through participating in co-design initiative. For example, co-researchers have expressed interest in publishing academic work and contributing to the co-design of the website.

Throughout the process thus far we have encountered challenges. Given the complexity of the phases and mixed responsibilities of various co-researchers across multiple institutions, HREC amendments represented a significate burden for university researchers. However, given the established need of this research, the complexity and related workload burden was justified within the research team. Furthermore, given the complexity of each of the school's individual term schedules and taking into account the individual schedules of each of the co-researchers it has been challenging to finding a suitable and continuing time to meet with co-researchers. These challenges are frequently reported for co-design research processes with adolescents [see (28)] and have been addressed by sharing responsibilities across the research team and conducting small group meetings at individual schools.

Student co-researchers have had significant input into the lesson development, supplemented by the additional quantitative and qualitative data that has been collected from their peers. Whilst the intervention is still to be piloted, the current draft lesson plan differs from the initial program that was first conceptualized by the university researchers. For example, there is a strong emphasis on providing learners with knowledge and skills that will enable them to identify and foster healthy relationships and optimize sexual wellbeing, as opposed to focusing primarily on the prevention of sexual violence. The student co-researchers have expressed strong opinions regarding preferred facilitators and provided contemporary vignettes that can be used as case study examples. They have also requested that the program helps them to develop healthy and respectful friendships, alongside romantic or sexual relationships. The importance of role playing and practicing awkward or uncomfortable conversations is a significant area of focus. Further refinements are expected during the piloting phase, but the formative process has already highlighted the positive benefits of a co-design process.

## Discussion

Employing a PAR approach within the sexuality education space is a novel approach, even more so when considering the limited use of co-design approaches undertaken related to sexual and reproductive health for adolescents and young people within Australia [see (21)]. Furthermore, these populations often have little to no influence in the messaging and education that surround CSE and adult voices dominate (8, 9).

Current CSE is felt to be outdated, negatively framed, and does not address the expressed needs of young people **(**11, 12). The proposed framework of engagement with co-researchers will allow for the creation and development of a positively framed CSE program, that is both contextually relevant and considered from the perspective of the adolescent end user. Furthermore, we feel as a team of adult researchers, that engagement with adolescents will provide further insight into the abilities of young people (29) to provide meaningful input in a CSE space, in which youth voice is lacking.

The research team is not naïve to the challenges related to undertaking participatory research involving adolescents and young people, such as ethical and logistical considerations. As such, practical solutions applied to mitigate the challenges will be documented and shared in subsequent publications. Although ethically when engaging with adolescents, working with parents is beneficial, there is potential that this impedes active involvement by the adolescents (25, 28) with the required ethical process inadvertently inhibiting youth agency (30). There can be tension between young people's interest and perceived benefits of participating in the research and that of the parent/guardian, whereby the parent/guardians can withhold consent **(**31, 32). Given the ongoing nature of the project, this may be the case, given we are designing a CSE program specifically for adolescents and young people and parents/guardians may have differing perspectives on what is considered appropriate for young people in the CSE space [see (33)]. However, recent national data from Australia does indicate that parents/guardians are highly supportive of CSE provision in schools (34).

This may also be a consideration when approaching schools. It may be the case there are restrictions in what schools will and will not allow participation, in as it has been acknowledged school administrations can act as gatekeepers to participation (35). This is despite the fact that an enabling school environment is known to be a key driver in the success of CSE programs (36).

Furthermore, although the value of youth voice has long been acknowledged in the field of PAR and YPAR as a key research practice when seeking to address youth-related topics [see (2, 6, 21)], the realities of undertaking this work is acknowledged. Although we have strived to create an authentic participatory co-design framework at the highest possible level, we have encountered challenges in the process to engaging in the highest level of participatory action. Like, Hart (37), the research team strive to ensure that the ability of young people to contribute is recognized and valued, while their time burden is minimized.

To conceptualise the current level of co-researcher participation at the time this paper is written, our engagement framework oscillates between rung five, *Consulted and Informed* and rung six, *Adult Initiated, Shared Decisions with Children* of Hart's (3) model of youth participation. Rung five, *Consulted and Informed,* posits that young people participate in the form of consultants, in a program designed and primarily run by adults, however young people participate in a way that ensures great integrity and their contributions are taken seriously. *Consulted and Informed* is still acknowledged as a level of participation, rather than non-participation (3). Rung six, *Adult Initiated, Shared Decisions with Children* posits even though the project is initiated by adults, the decision-making responsibilities are shared with young people (3). There have been moments where student co-researchers were consulted, and their feedback has led to changes in the approach taken.

For example, the student co-researchers have clearly articulated preferred characteristics of prospective facilitators. The feedback received from student co-researchers reframed the delivery plans, to ensure that facilitators during the pilot phase will be independent from the school, knowledgeable about the content area, and relatively young in age. Student co-researchers have also provided specific direction regarding the delivery mode, lesson structure and case study vignettes. Whilst university researchers are actively striving to engage in true and authentic co-design and redistribution of power, the reality is that this is not always possible. However, as noted by Hart (37), the participation of young people should consider what is appropriate at the time, and full participation is not necessarily required.

Thus far, the intentional inclusion of student co-researchers early during intervention conceptualization and the iterative nature of the framework has allowed potential challenges to be recognized and considered across all phases of the project. However, the importance of maintaining flexibility and adaptability within the participatory process is also acknowledged (38). In contrast to contemporary discourse that CSE can be negatively framed, the current engagement framework has led to the drafting of an intervention that seeks to optimize interpersonal skills and sexual wellbeing, and it will now transition to the piloting phase. All members of the research team (university researchers, student co-researchers, school staff) consider the flexibility and adaptability of this framework to be a methodological design strength.

## Data Availability

The original contributions presented in the study are included in the article/Supplementary Material, further inquiries can be directed to the corresponding author/s.
